# Factors associated with cytomegalovirus infection in children undergoing allogeneic hematopoietic stem-cell transplantation

**DOI:** 10.1097/MD.0000000000014172

**Published:** 2019-01-25

**Authors:** Tang-Her Jaing, Tsung-Yen Chang, Shih-Hsiang Chen, Yu-Chuan Wen, Ting-Jiuan Yu, Ching-Fen Lee, Chao-Ping Yang, Pei-Kwei Tsay

**Affiliations:** a Division of Hematology/Oncology, Department of Pediatrics, Chang Gung Children's Hospital; b Department of Nursing; c Division of Clinical Pharmacy, Department of Pharmacy, Chang Gung Memorial Hospital; d Department of Public Health and Center of Biostatistics, College of Medicine, Chang Gung University, Taoyuan, Taiwan.

**Keywords:** allogeneic hematopoietic stem-cell transplantation, cytomegalovirus, pediatric, reactivation

## Abstract

While preemptive therapy with ganciclovir (GCV) for cytomegalovirus (CMV) infection is used following allogeneic hematopoietic stem-cell transplantation (HSCT), risk factors for CMV infection in children undergoing HSCT are poorly understood.

We studied CMV reactivation following allogeneic HSCT by retrospectively analyzing pediatric patients who received allogeneic HSCT and preemptive GCV therapy between 1998 and 2016. The level of viremia requiring preemptive GCV therapy was >1 CMV antigen-positive cells per 5 × 10^5^ leukocytes during the antigenemia assay era and >1000 copies/mL in the polymerase chain reaction era. Among 290 at-risk patients, 54 (18.6%) patients had primary CMV infection or CMV reactivation occurring at a median of 76 days (range, 7–234) following HSCT. CMV reactivation occurred in 28.2% (44/156) of CMV-seropositive transplant recipients at a median of 26 days posttransplant.

Univariate and multivariate analyses revealed statistically significant relationships between CMV infection and grade III–IV acute graft-vs-host disease, seronegative donor/seropositive recipient combination, and unrelated/mismatched donors. The remaining demographic factors were not predictive of CMV infection.

The seronegative donor/seropositive recipient combination for HSCT was associated with an incomplete response to antiviral therapy. Human leukocyte antigen identical donors were the best choice for patients undergoing allogeneic HSCT to reduce the incidence of CMV disease and mortality.

## 1 Introduction

Cytomegalovirus (CMV) is recognized as an important cause of morbidity and mortality in recipients of hematopoietic stem-cell transplants (HSCTs). Prophylaxis and preemptive therapy are used to prevent CMV disease in patients undergoing HSCT. Although preemptive therapy is the most commonly used approach, some clinicians favor prophylactic therapy for high-risk patients such as CMV-negative donor CMV-positive recipient serostatus, cord blood products, unrelated or mismatched donor.^[1–3]^ Quantitation of CMV DNA using real-time polymerase chain reaction (PCR) is utilized for monitoring CMV infection.

While the CMV antigenemia assay remains the gold standard,^[4]^ cutoff values for the number of CMV antigen-positive cells required to initiate ganciclovir (GCV) therapy have not been clarified.^[5]^ Current strategies for mitigating the effect of CMV infection on outcomes include weekly measurement of CMV antigenemia or viral loads in peripheral blood through posttransplant day 100 and initiation of acyclovir prophylaxis in the peritransplant period in patients at risk for CMV infection.

Because of the inconclusive results and weaknesses of previous studies as well as potential risks associated with early and long-term use of GCV in this high-risk patient population, we investigated risk factors contributing to treatment failure in pediatric patients at high risk for CMV reactivation.

## 2 Methods

### 2.1 Patients, donor, and disease characteristics

We retrospectively reviewed the computerized database of Chang Gung Children's Hospital for all patients aged 0 to 20 years who underwent allogeneic HSCT between April 1998 and December 2016. Immunodeficient patients who were treated with intravenous immunoglobulin (IVIg) during pretransplantation and those treated for CMV infection before HSCT were excluded from this study. Informed consent was waived by the institution review board of Chang Gung Children's Hospital because of the retrospective nature of this study.

### 2.2 Study definitions

All patients were routinely screened for CMV viremia at 1-week intervals until 50 days posttransplant, biweekly until 100 days posttransplant, and subsequently on follow-up whenever indicated clinically until 1 year posttransplant. Reactivation was defined as positive antigenemia (>1 CMV antigen-positive cell per 5 × 10^5^ leukocytes), or elevated CMV DNA levels (>1000 copies/mL). CMV infection was defined as isolation of the CMV or detection of viral proteins or nucleic acids in any body fluid or tissue specimen. CMV disease was defined by the presence of signs and/or symptoms of end-organ disease along with CMV detection in related fluid or tissue samples.^[6]^ All cord blood units were considered seronegative. Monitoring was initiated after neutrophil engraftment using CMV antigenemia or plasma-based PCR assay and continued through day 100 posttransplant. Patients with positive CMV antigenemia or elevated CMV DNA levels by quantitative PCR were monitored weekly until normalization.

Intravenous (IV) acyclovir followed by oral acyclovir and early IVIg administrations were recommended for all seropositive patients at our institution. CMV reactivation was preemptively treated with GCV as follows: induction with 5 mg/kg IV GCV twice daily for 2 weeks, followed by maintenance with 5 mg/kg IV GCV daily for an additional 6 weeks. Recommendations for IVIg replacement applied to all patients undergoing allogeneic HSCT.

### 2.3 CMV antigenemia assay

The CMV antigenemia assay was performed within 4 hours of specimen collection using the MONOFLUO CMV kit (Bio-Rad, Marnes-la-Coquette, France). This assay utilizes monoclonal antibodies to detect CMV matrix phosphoprotein pp65, a late structural protein expressed in blood leukocytes during the early phase of the CMV replication cycle. Briefly, antigenemia was measured by immunoflurescence quantitation of pp65-positive leukocyte nuclei using cytospin preparations of 5 × 10^5^ peripheral blood leukocytes.

### 2.4 Quantitation of CMV DNA in plasma by real-time PCR

Real-time PCR of plasma samples was performed to detect the highly conserved, nondrug target region of the CMV DNA polymerase (UL54) gene. Standard silica-based specimen preparation methods were used to capture CMV DNA and CMV quantitative standard (QS) DNA, and defined oligonucleotides were used as primers for amplification of CMV DNA and CMV QS DNA. Briefly, CMV DNA was isolated from 350 μL EDTA plasma samples using the COBAS AmpliPrep/COBAS TaqMan CMV Test (Roche, Basel, Switzerland). The clinical significance of low CMV DNA levels (<100–500 copies/mL) may be difficult to interpret, particularly in whole blood samples, as low CMV DNA levels in whole blood or plasma do not always correlate with disease development. Therefore, the cutoff for viremia prompting preemptive therapy was >1 CMV antigen-positive cell per 5 × 10^5^ leukocytes in the antigenemia assay era and ≥1000 copies/mL in the PCR era.

### 2.5 Statistical analysis

Statistical analyses were performed using SPSS version 20 (SPSS Inc, Chicago, IL). We set up 10 parameters for univariate analysis (Table 1) and analyzed the relationship between CMV infection and these parameters by logistic regression. Associations between determinants and CMV seroprevalence were analyzed using univariate analysis. Determinants with a *P*-value <.05 on univariate analysis were included in the logistic regression model. Continuous variables between the 2 groups were performed using the nonparametric Mann–Whitney *U*-test, and categorical variables were compared using the Chi-squared or Fisher exact test, as appropriate. All descriptive statistics calculated for the categorical variable were reported as percentages, and continuous variables were presented as medians. CMV antigenema and CMV DNA viral loads were log-converted to fit for parametric analyses. Differences in numerical variables between the 2 groups were assessed by Welch *t*-test. Variables with *P*-value <.10 by univariate analysis were included in multivariate logistic regression analysis. *P* < .05 was considered statistically significant.

**Table 1:**
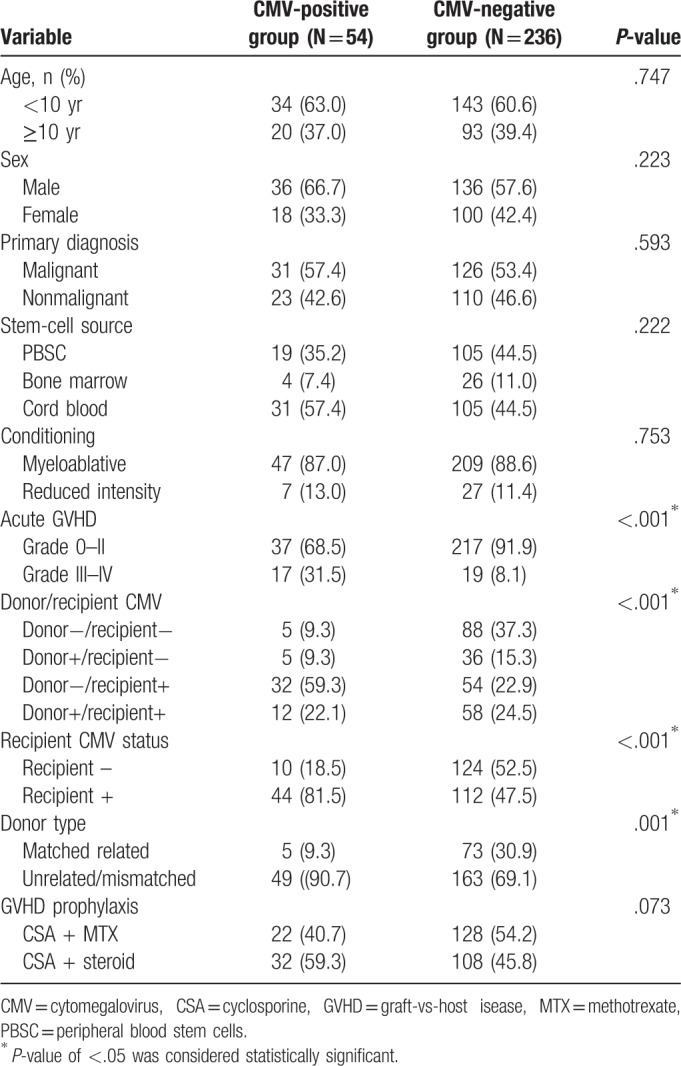
Univariate analysis of risk factors for CMV reactivation/infection in pediatric patients (n = 290).

## 3 Results

This single-center, retrospective study included 290 patients who received allogeneic HSCT with bone marrow (n = 30), peripheral blood (n = 124), and umbilical cord blood (n = 136). Conditioning therapy comprised a myeloablative regimen (n = 256) or reduced-intensity conditioning (n = 34). The donor stem-cell sources included 78 human leukocyte antigen (HLA)-identical siblings, 203 unrelated donors, and 9 haploidentical matched parental stem cells.

Among 290 at-risk patients, 54 (18.6%) patients had primary CMV infection or CMV reactivation occurring at a median of 76 days (range, 7–234) following HSCT. CMV reactivation was detected in 44 (28.2%) of 156 CMV-seropositive transplant recipients at a median of 26 days posttransplant. IV GCV was ultimately administered as preemptive therapy to 50 patients. Moreover, hyperimmune CMV immunoglobulin was administered in 10 of these 50 patients. One patient presented with a significantly higher CMV viral load and subsequently developed acute interstitial pneumonitis requiring rescue extracorporeal membrane oxygenation. He eventually succumbed to respiratory failure secondary to the possible emergence of CMV resistance.

Univariate analyses revealed statistically significant relationships between CMV infection and grade III–IV acute graft-vs-host disease, seronegative donor/seropositive recipient combination, and use of unrelated/mismatched donors. Multivariate logistic regression analysis showed that these 3 variables were independent risk factors for CMV infection (Table 2). The remaining demographic factors included in the analysis were not predictive of CMV infection. These findings indicated that seronegative donor/seropositive recipient combination for HSCT was associated with an incomplete response to antiviral therapy. However, the risk of CMV infection was not increased in recipients of cord blood compared to those of stem cells from other sources.

**Table 2:**
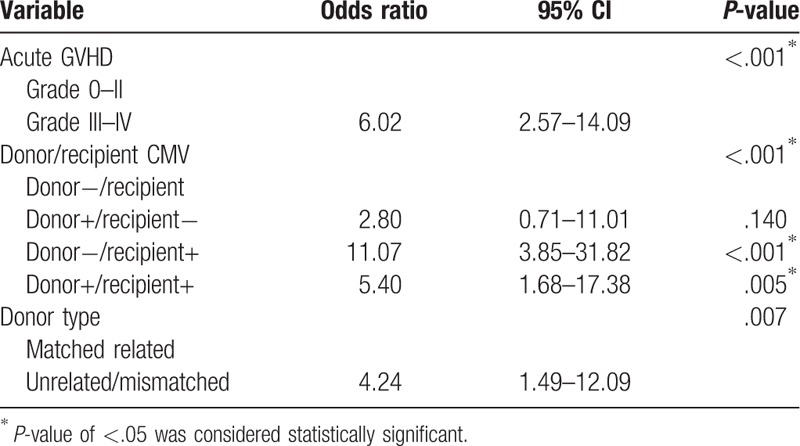
Multivariate analysis of risk factors for CMV reactivation/infection.

## 4 Discussion

A majority of CMV infections are caused by virus reactivation, which usually occurs within 3 months following transplantation.^[7–9]^ Risk factors predicting CMV infection in pediatric patients undergoing HSCT are poorly understood, and factors involved with the recurrence of CMV viremia following an initial episode have not been reported.^[10]^ Two methods have been utilized to diagnose CMV infection: the CMV antigenemia assay to detect pp65 and PCR to detect CMV DNA. Both techniques can be used for early detection of CMV replication.^[11]^ The antigenemia assay is likely to be replaced with molecular methods, particularly for monitoring CMV replication after transplantation.^[12,13]^ The present study featured a moderate CMV infection rate (22.8%), but patients with cord blood transplantation (CBT) did not show a statistically significant association with CMV reaction, despite a crude trend in increased numbers of CMV infection among CBT than adult stem-cell transplantation.

The incidence of CMV primary infection or reactivation in the present study was 18.6%, which was lower than the rate in historical controls (30%).^[9]^ Molecular diagnostic methods have replaced or supplemented traditional methods, such as viral culture and antigen detection, for diagnosis of viral infections. As compared with the CMV antigenemia testing, quantitative PCR was associated with earlier detection of CMV infection. Although quantitative PCR for CMV has replaced antigenemia for routine monitoring, until recently, inter-laboratory correlation of viral loads was poor due to lack of an international standard.^[14]^ Higher maximum CMV PCR titers, although not available for every patient, trended toward significance in univariate analysis.^[15]^ However, a CMV DNA load of >1000 copies/mL after 2 weeks of GCV treatment was reported to be suggestive of drug resistance.^[16]^

The CMV antigenemia assay is a labor-intensive and low-throughput method, which is not amenable to automation. Patient samples must be processed within 6 hours, as delays were shown to significantly reduce the assay sensitivity.^[17]^ Specimen deterioration with time after sample collection is a lesser concern in PCR-based assays than in other tests for CMV detection.^[18,19]^ Allogeneic HSCT can potentially reduce the high relapse rate for patients with leukemia; however, CMV reactivation following HSCT should be addressed to improve patient outcomes.

This study has several limitations, most of which related to its retrospective design. The patients were treated with different protocols during the study period. Additionally, donors included both HLA-identical and alternative donors (including haploidentical transplantation). The leukocyte-based CMV antigenemia assays were positive at an earlier time after HSCT and became negative more rapidly after initiation of GCV therapy than the plasma-based PCR assays.^[20,21]^ GCV therapy is a relevant, clinically tailored treatment for patients with CMV reactivation. Further studies are required to determine the appropriate timing of preemptive antiviral strategies to improve CMV infection.

## Author contributions

**Conceptualization:** Tang-Her Jaing, Shih-Hsiang Chen.

**Data curation:** Tsung-Yen Chang, Yu-Chuan Wen, Ting-Jiuan Yu.

**Formal analysis:** Tsung-Yen Chang, Yu-Chuan Wen, Pei-Kwei Tsay.

**Funding acquisition:** Tang-Her Jaing.

**Investigation:** Shih-Hsiang Chen, Ching-Fen Lee.

**Methodology:** Ching-Fen Lee.

**Software:** Pei-Kwei Tsay.

**Supervision:** Chao-Ping Yang.

**Validation:** Pei-Kwei Tsay.

**Writing – original draft:** Tang-Her Jaing.

**Writing – review & editing:** Tang-Her Jaing, Shih-Hsiang Chen.
